# Exposure, Environment, and Well Being. A Cross-Sectional Study of the Health Hazards, the Working Environment and the Quality of Life Among Cashew Workers in South Kerala, India

**DOI:** 10.1007/s10900-025-01524-z

**Published:** 2025-10-16

**Authors:** Mary Dello Rebello, Betsy A Jose, Manjula Anil Kunder, Afraz Jahan, Felix Johns, Akshay Kumar

**Affiliations:** 1 Department of Community Medicine, Kasturba Medical College, Manipal, Manipal Academy of Higher Education, Manipal, Udupi, Karnataka 576104 India; 2 Department of Community Medicine, Pushpagiri Institute of Medical Science and Research Center, Thiruvalla, Kerala 689101 India; 3 Department of Hospital Administration, Kasturba Medical College, Manipal, Manipal Academy of Higher Education, Manipal, Udupi, Karnataka 576104 India

**Keywords:** Cashew workers, Industry, Quality of life, Musculoskeletal pain, Occupational health, Working conditions

## Abstract

Cashew processing is a labor-intensive work, which requires physical demand exposing workers to various occupational health problems. This study aims to identify the occupational health issues faced by cashew workers, evaluate their working environment and assess their quality of life. A cross-sectional study was conducted among 280 cashew factory workers in South Kerala. Data was collected using a semi structured questionnaire which includes sociodemographic characteristics, working environment, and system related health issues. The WHOQOL-BREF questionnaire was used to assess Quality of life among workers. The collected data was entered and coded into Microsoft Excel (Version 2016) and analyzed via SPSS Version16.0. Prevalence of musculoskeletal pain (96.8%) was high, followed by central nervous system-related symptoms (68.2%) and dermatological issues (58.5%).Of the ten factories surveyed, one-third (33.3%) did not have a proper system for fumes and dust extraction, provision of personal protective equipment, appointment of welfare officers and accessible washing facilities. Participants above 45 yrs of age were found to have 1.7 times higher odds of experiencing occupational health issues compared to those aged less than 45 years, as per multivariate analysis(*p* < 0.05). With respect to Quality of life, workers had higher social health mean score of 65.11 ± 15.02 and lower psychological health mean score of 46.93 ± 10.61. Musculoskeletal pain was the most prevalent health issue among cashew workers. The working environment among cashew workers is generally poor, which, along with occupational hazards, contributes to compromised quality of life in multiple domains.

## Introduction

 Cashew nuts were previously recognized as rich man’s food and poor man’s crop, as they are cultivated extensively in underdeveloped and developing countries and later exported to developed countries [1, 2]. India exports cashew kernels to more than 60 counties globally [3]. Kerala stands fifth in cashew processing units, and private firms dominate the global market [4–6]. Since there are numerous cashew processing units and factories in the Kollam district, where many individuals are employed, it was previously known as the cashew capital of the country [5].Cashew processing is labor-intensive work, and the Indian industry plays a significant role in promoting employment opportunities, thereby sustaining livelihoods for many cashew workers [4, 7, 8]. Health and financial well-being benefit only if workers are employed under favorable conditions. Certain environmental factors in cashew processing factories expose workers to various health hazards, such as injuries, respiratory diseases and other health complications. The processing of cashew involves certain phases, such as roasting, shelling, peeling and grading [9]. Every phase involves a certain amount of ergonomic risk, including exposure to furnace smoke and burns during roasting, damage to the skin due to contact with cashew nutshell liquid during shelling, improper sitting positions that result in joint and back pain, repetitive movements during shelling and peeling, and poor body positioning during nearly every task involving cashew processing [8, 10].

Cashew workers are characterized predominantly by low socioeconomic status, which heightens their susceptibility to income volatility [11]. Low earnings and the economically disadvantaged circumstances of workers’ families further deprive them of their well-being [12]. Quality of life is a multidimensional concept and is influenced in a complex manner by various factors, including physical health, psychological wellbeing, social relationships and interactions with the environment [13, 14]. Numerous studies have focused on occupational health hazards; only limited research has been conducted specifically to assess the work environment in the cashew industry and quality of life among cashew workers. Kollam, which is a major center for cashew processing and employs a significant workforce in the industry, serves as a representative case for understanding the dynamics of the cashew sector in India. Therefore, conducting a study in these districts holds national relevance. The objective of this study was to identify the occupational health-related problems faced by cashew workers, to evaluate the working environment and to assess the quality of life among cashew workers in the Kollam district, Kerala.

## Methods

### Study Design and Participants

A cross-sectional study was conducted in ten cashew processing factories in Kollam District in Kerala from August 2021 to August 2022. Full-time cashew industry workers aged 18 to 70 years, with over one year of employment, were included. Individuals with known systemic diseases involving musculoskeletal system such as rheumatoid arthritis, Systemic Lupus Erythematosus, Mixed Connective Tissue Disease and Sjogren’s syndrome; with known case of pulmonary fibrosis, sarcoidosis, cystic fibrosis, tuberculosis, pneumoconiosis and also neuromuscular lung disease such as those caused by neuromuscular disease and amyotrophic lateral sclerosis; with known cases of skin diseases such as psoriasis, vitiligo, ichthyosis, measles, seborrheic dermatosis, lupus, chicken pox and impetigo; with tooth decay and abrasion of teeth; with known pre-existing psychiatric disorder such as schizophrenia, bipolar disorder and severe depression with psychosis were excluded.

Here, a *cashew factory* is defined as a place where processing of cashew nuts is carried out and includes the factory building, the site thereof, and the land nearby the factory, which is necessary for, or in connection with, the working of the factory. *Full-time workers* were defined as those workers who work for a minimum of 4 days per week, i.e., 20 days per month with an eight-hour duration of work per day, and those who work for a minimum of 150 days per year [5].

### Sample Size Estimation and Sampling Procedure

We estimated a sample size of 280 participants by using the formula [Z _(1−α/2)_) [2] p (1-p)/d^2^] × D eff.

Assuming that the proportion of workers with musculoskeletal pain (p) was 30% according to a previous study [15], the precision (d) was 20% of p with a 95% confidence interval (CI), with a design effect of 1.25. We used multistage cluster sampling by listing the factories from the cashew welfare board and then stratified them into government and private. Two corporations and eight private factories were selected via probability proportional to size sampling at a ratio of 1:4. Factories were selected via simple random sampling. Stratification of the list of employees obtained from each factory was performed based on the work process. Furthermore, seven individuals were selected from each group via simple random techniques. According to the Factories Act, women and adolescents are not permitted to clean, lubricate or adjust any part of the machine or equipment in motion. Since roasting requires machinery work, only males are engaged in the process [16, 17]. Hence, in our study, we included men and women at a ratio of 1:4.

Face‒to-face interviews were conducted via a semi structured questionnaire. Ethics clearance for the study was obtained from the institutional ethics committee. Written informed consent was obtained from all participants prior to their inclusion in the study.

### Data Collection Tools

The questionnaire was originally developed in English, translated to the local language and validated before data collection began. The final structured interview consisted of five parts.

- *Sociodemographic characteristics* include age, sex, education, education of the head of family, nature of work, work status, occupation of the head of family, monthly income, daily income, marital status, type of housing and number of family members.
- *Health conditions potentially associated with occupational exposure* –.
  Participants self-reported health complaints experienced over the past one year were noted and were subsequently categorized based on the affected body system. Selected clinical parameters, including body mass index, random blood glucose and systolic and diastolic blood pressure, were objectively measured and recorded during the visit.
- *Workplace safety* was evaluated using guidelines from factories act [16].
- *Quality of* life was measured via the WHOQOL-BREF scale, Using one standard deviation below the mean as cut off point for defining low quality of life [17].

### Statistical Analysis

The data was entered into Microsoft Excel version 2016 and coded accordingly. The data were analyzed via SPSS software version 16. Descriptive statistics for sociodemographic characteristics, working conditions, and occupational health hazards were summarized using frequencies and percentages. The associations between occupational health-related problems and the nature of work were examined via the chi-square test. Binary logistic regression was performed to assess the relationships between sociodemographic characteristics and occupational health-related problems, with a focus on variables with p values less than 0.3 in the unadjusted analysis. The adjusted odds ratio was calculated with a 95% confidence interval, and a p value of less than 0.05 was considered statistically significant. Domain scores for quality of life were calculated individually, and transformed scores were derived from the raw scores.

## Results

### Study Participants’ Characteristics and Working Conditions

A total of ten factories were surveyed and assessed for their working environment, and occupational health-related problems and quality of life were assessed among 280 cashew workers. Among the 280 cashew workers, 210 (75%) were females, and 70 (25%) were males. The mean age of the study participants was 45.7 ± 12.1 years. Almost half of them had completed high school (*n* = 115, 41.1%), and the majority were married (*n* = 222, 79.3%). The majority of them (*n* = 259, 92.5%) had low socioeconomic status. Nearly half of the participants had a minimum of 20 years of work experience. Table 1 shows that while all cashew factories provided basic facilities such as toilets and waste treatment, overall compliance with occupational health and safety standards was inadequate. Key provisions such as personal protective equipment, dust and fume control, washing facilities, and creches were available only in a few units. Lighting, ventilation and welfare measures have been inconsistently implemented, highlighting significant gaps in worker safety and welfare. Interestingly, none of the factories employed child or adolescent workers. Workers involved in roasting at private firms typically work only 3 to 4 days a week. In contrast, workers in other processing sections generally work 5 to 6 days a week.

**Table 1:** Sociodemographic characteristics and working environments of the study participants(*n* = 280)

Characteristics	Frequency	Percentage
*Age (in years)*		
21–30	47	16.8
31–40	39	13.9
41–50	73	26.1
51–60	102	36.4
61–70	19	6.8
*Educational status*		
Illiterate	16	5.7
Primary school	70	25.0
Middle school	61	21.8
High school	115	41.1
Intermediate	18	6.4
*Marital status*		
Unmarried	22	7.9
Married	222	79.3
Divorced/Widow	36	12.8
*Type of family*		
Nuclear	222	79.3
Joint	37	13.2
Extended	21	7.5
*Type of housing*		
Kutcha	22	7.9
Pucca	89	31.8
Semi pucca	169	60.4
*Modified Kuppuswamy classification 2022*		
Lower middle	4	1.4
Upper lower	259	92.5
Lower	17	6.1
*Permanent workers with respect to nature of work (n = 139)*		
Roasting	14	10.1
Shelling	41	29.5
Peeling	44	31.7
Grading	40	28.8
*Temporary workers with respect to nature of work (n = 141)*		
Roasting	56	39.7
Shelling	29	20.6
Peeling	26	18.4
Grading	30	21.3
*No. of years of work experience*		
1–10	109	39
11–20	73	26.1
21–30	54	19.3
31–40	39	13.9
41–50	5	1.8
*Working environment (n = 10 factories)*		
Treatment for waste and effluents, and for their disposal	10	100
Availability of toilet facilities	10	100
Cleanliness of working place	8	83.3
Precaution against dangerous fumes	8	83.3
Availability of first aid appliances	8	83.3
Provision for control of temperature	8	83.3
Availability of lunchroom facilities	8	83.3
Facility for storing and drying clothes	8	83.3
Employee for keeping the toilets clean	8	83.3
Adequate lighting	6	66.7
Adequate ventilation	6	66.7
Provision for seating facilities	5	50
Provision for eliminating fumes and dust	2	33.3
Provision of personal protective equipment while doing work	2	33.3
Supply of drinking water facility	2	33.3
Availability of washing facilities	2	33.3
Employment of welfare officer	2	33.3
Availability of crèches facilities	2	33.3
Provision of leave with wages	2	33.3
Availability of canteen services	0	0
Employment of adolescent/child workers	0	0

Among the participants, a significant prevalence of various health problems was observed, with musculoskeletal pain being the most common, affecting 96.5% of workers. The most frequently reported musculoskeletal complaints were low backache (56.8%) and knee pain (49.3%) (Table 2).

**Table 2:** Distribution of study participants based on their self-reported health problems (*n* = 280)

Characteristics		
*Central Nervous System (n = 191)*	Frequency	Percentage
1. Syncopal Attack	63	22.5
2. Headache	154	55
*Ophthalmology*		
1.Eye Irritation/Watering of Eyes	110	39.3
*Ear Nose Throat*		
1. Hearing Problems	20	7.1
Oral (*n* = 72)		
1. Difficulty in Opening the Mouth	9	3.2
2. Recurrent Ulceration in Mouth	21	7.5
3. Dryness of Mouth and Burning Sensation in Oral Cavity	57	20.4
*Dermatological (n = 164)*		
1.Skin Irritation/Redness	66	23.6
2. Black Pigmentation on Hands and Feet	47	16.8
3. Itching due to dust particles/due to bites of insects while work	29	10.4
4. Infected Nails	39	13.9
5. Thin and tapered nails after started working in the industry	26	9.3
6. Burns while roasting cashews	36	12.9
7. Injury/trauma while crushing cashews	43	15.4
*Vascular*		
1.Swollen blood vessels/discoloration on the skin of your legs	32	11.4
*Respiratory*		
1. Difficulty in breathing	69	24.6
*Genitourinary and Anal (n = 79)*		
1. Dark colored urine	32	11.4
2. Genital Issues	19	6.8
3. Piles	44	15.7
*Musculoskeletal Pain (n = 271)*		
*Site of pain*		
*Neck*	76	27.1
*Shoulders*:		
1. Right shoulder	155	55.4
2. Left shoulder	100	35.7
3. Both shoulder	78	27.9
*Elbow*	62	22.1
Wrist/hand:		
1. Right wrist/hand	70	25
2. Left wrist/hand	51	18.2
3. Both wrist/hand	46	16.4
*Upper back*	53	18.9
*Lower back*	159	56.8
*One or both hip/thigh*	30	10.7
*One or both knee*	138	49.3
*One or both ankle/feet*	51	18.2

The health status of the study participants was assessed, and half of the participants were found to be prehypertensive, followed by stage 1 hypertension (*n* = 67,23%) and stage 2 hypertension (*n* = 43,15.4%), according to the JNC 8 classification. Only a few participants were found to be in diabetic range (*n* = 4, 1.4%), followed by those in prediabetic range (*n* = 20, 7.1%). With respect to nutritional status, more than one quartile of the study participants were obese (*n* = 96, 34.3%), followed by overweight and underweight participants, whose proportions were the same (*n* = 17, 6.1%).

### Relationships with Work Type and system-related Involvement

The data in Table 3 demonstrate significant variations in the prevalence of health issues among workers involved in different sections of cashew processing units. Among the health problems, CNS, ophthalmologic and musculoskeletal clinical presentations were found to be statistically significant with the nature of work.

**Table 3:** Association of system-related problems with the nature of work

System related health problems	Roasting (*n*, %)	Shelling (*n*, %)	Peeling (*n*, %)	Grading (*n*, %)	Total	ꭓ2	*P* value
*Central nervous system*							
Absent	31 (44.3)	14 (20.0)	25 (35.7)	19 (27.1)	89	10.72	0.013
Present	39 (55.7)	56 (80.0)	45 (64.3)	51 (72.9)	191		
*Ophthalmology*							
Absent	53 (75.7)	38 (54.3)	41 (58.6)	38 (54.3)	170	9.164	0.027
Present	17 (24.3)	32 (45.7)	29 (41.4)	32 (45.7)	110		
*Ear*							
Absent	69 (98.6)	62 (88.6)	66 (94.3)	63 (90.0)	260	6.897*	0.073
Present	1 (1.4)	8 (11.4)	4 (5.7)	7 (10.0)	20		
*Respiratory*							
Absent	50 (71.4)	53 (75.7)	51 (72.9)	57 (81.4)	211	2.21	0.530
Present	20 (28.6)	17 (24.3)	19(27.1)	13 (18.6)	69		
*Dermatological*							
Absent	24 (34.3)	24 (34.3)	34 (48.6)	34 (48.6)	116	5.89	0.117
Present	46 (65.7)	46 (65.7)	36 (51.4)	36 (51.4)	164		
*Oral cavity*							
Absent	59 (84.3)	52 (74.3)	50 (71.4)	47 (67.1)	208	5.83	0.120
Present	11 (15.7)	18 (25.7)	20 (28.6)	23 (32.9)	72		
*Vascular*							
Absent	65 (92.9)	61 (87.1)	60 (85.7)	62 (88.6)	248	1.98	0.577
Present	5 (7.1)	9 (12.9)	10 (14.3)	8 (11.4)	32		
*Genitourinary and anal system*							
Absent	61 (87.1)	46 (65.7)	50 (71.4)	44 (62.9)	201	12.19	0.07
Present	9 (12.9)	24 (34.3)	20 (28.6)	26 (37.1)	79		
*Musculoskeletal* Pain							
Absent	6 (8.6)	2 (2.9)	1 (1.4)	-	9	7.64*	0.034
Present	64 (91.4)	68 (97.1)	69 (98.6)	70 (100)	271		

*Fisher’s exact test

Table 4 shows that in the univariate analysis, age, sex, the nature of work and the number of years of work were significantly associated with multiple occupational health issues. Multivariate analysis of all the variables which were significant in univariate revealed that only age was significantly associated with multiple occupational health issues. Workers aged more than 45 years experienced health problems 1.7 times more often than those aged less than 45 years did (p value = 0.04).

**Table 4:** Determinants of occupational health-related problems among study participants

Characteristics	Sub category	Presence of Multiple Occupational health problems*	OR (95%CI)	*P* value	Adjusted OR (95%CI)	*P* Value
Age Category	21–45	42 (32.1)	Ref	0.001	Ref	0.044
	46–70	89 (67.9)	2.328 (1.429–3.792)		1.777 (1.017–3.105)	
Sex	Females	108 (82.4)	2.164 (1.227–3.816)	0.007	1.004 (0.466–2.165)	0.992
	Males	23 (17.6)	Ref		Ref	
Nature of work	Roasting, Grading	55 (42.0)	Ref	0.012	Ref	0.246
	Shelling, Peeling	76 (58.0)	1.835 (1.141–2.951)		0.703 (0.388–1.274)	
No of years of work	< 15years	55 (42.0)	Ref	0.001	Ref	0.062
	> 15years	76 (58.0)	2.230 (1.381–3.601)		1.683 (0.974–2.906)	

*The presence of more than 3 system-related health issues was considered multiple occupational health problems,* and the presence of fewer than 3 system-related health issues was considered the absence of multiple occupational health problems.*

Table 5. shows the scores in all four domains as well as overall quality of life and general health. Among the domain, the social health domain had the highest mean score(65.11 ± 15.02), while psychological domain had the lowest (46.93 ± 10.61).Using two standard deviation below the mean as cut off point for defining low quality of life, it was found that 62.9% had poor QOL in phycological domain, followed by 53.9% in environmental domain,23.2% in physical domain and 11.4% in social relationships domain. Using correlation coefficient, moderately positive correlation was seen between physical and general health(r = + 0.420:*p* < 0.001),physical and psychological quality of life(r = + 0.444:*p* < 0.001).Similarly, physical and environmental QOL(*r* = 0.326:*p* < 0.001),psychological and environmental QOL(*r* = 0.346:*p* < 0.001) and environmental and overall QOL(*r* = 0312:*p* < 0.001). However, a low negative correlation was observed between social, environmental and overall quality of life.

**Table 5:** Spearman’s rank correlations of WHOQOL -BREF domains, overall quality of life and general health (*n* = 280)

Domains	Mean	SD	No of participants with poor score^a^, *n*(%)	General health	Overall quality	Environmental QOL	Social QOL	Psychological QOL	Physical QOL
Physical	54.61	9.50	65 (23.2%)	0.420**	0.277**	0.326**	0.204**	0.444**	1.00
Psychological	46.93	10.61	176 (62.9%)	0.233**	0.244**	0.346**	0.089**	1.00	0.444**
Social	65.11	15.02	32 (11.4%)	0.269**	− 0.0175**	−0.091**	1.00	0.089**	0.204**
Environmental	49.39	10.32	151 (53.9%)	0.121**	0.312**	1.00	−0.091**	0.346**	0.326**
Overall QOL	32.41	14.07	212 (75.7%)	0.177**	1.00	0.312**	−0.175**	0.244**	0.277**
General health	55.09	17.67	39 (13.9%)	1.00	0.177**	0.121**	0.269**	0.223**	0.420**

^a^Score <2SD

^**^p < 0.001

## Discussion

The present study revealed that workers involved in cashew processing units experience a range of health problems. Repetitive movements, prolonged physical strain and awkward positions often lead to musculoskeletal pain among shelling, peeling and grading sections. Certain tasks, such as scooping and lifting heavy sacks of raw cashews by workers in the roasting section, also face significant physical strain. Low backache is the most frequent issue reported from all processing sections in our study. Similarly, low back pain was consistently reported by Prasad, Nelson et al., and K. Madhura et al. [11, 18, 19]. Workers involved in shelling adapt squatting position to complete the task, the risks of prolonged sitting and repetitive tasks, leading to significant exposure to strain on the back, shoulders, wrists, and neck [3, 7, 9, 20].Half of the surveyed factories lacked seating facilities, forcing workers to sit on the floor, which can lead to musculoskeletal problems. Exposure to dust and fumes during roasting and shelling also contributes to respiratory issues. Around 30% of workers in roasting units reported respiratory issues in our study, inadequate working environment such as lack of ventilation, exhaust fans and proper protective measures could be the reason.

Dermatological issues were common, with more than half of the participants reporting skin problems, particularly burns in the roasting section. Shelling workers reported injuries to fingers hammering the shells and black pigmentation due to exposure to cashew nutshell liquid. Priya et al. reported that blackish staining does not cause severe destruction to the skin, and workers are habituated to it ^21^. Nair et al. reported that atopic dermatitis, contact urticaria and dry skin were prevalent among cashew workers [21]. Headaches were reported by more than half of the participants, likely resulting from the ongoing stress associated with meeting work demands. Malathi reported similar findings, with 69% of workers experiencing headaches under comparable conditions [22]. Ophthalmic problems, including eye strain, were prevalent in the shelling, grading and peeling sections because of constant visual demand. Studies conducted by Nelson et al., Priya et al., and Madhuri et al. reported similar findings [11, 19, 23]. Genitourinary problems were reported by 18.2% of the workers in our study, primarily in the shelling and grading sections. Heat exposure, contamination from cashew shell liquid, and infrequent urination due to work overload are contributing factors [5, 8]. Venugopal et al. reported that there is a 7.7-fold increased risk of urogenital symptoms among workers in similar settings [8].

In the present study, the survey of the working environment revealed that only two factories implemented measures to eliminate fumes and dust, provide crèche and washing facilities, employ welfare officers, and offer paid leave. Similar findings were observed in Sajeena study. Most participants in our study belonged to low socioeconomic backgrounds, with a higher representation of women in cashew processing units. This further emphasizes their dual burden of managing both household and work responsibilities, which heightens the risk of health issues further draining their energy [5, 23]. Similar to our study, Srinivasan R reported that workers, particularly women, were highly dissatisfied with their working conditions and faced poor living standards and socioeconomic challenges [4].

Workers experienced higher social QOL(mean score 65.11 ± 15.02) and lower psychological QOL (mean score 46.93 ± 10.61).The four domains, overall QOL and general health were significantly and positively interrelated with low to moderate relationships. Jayakumar reported that experienced workers exhibited stronger social relationships which correlates with the current study [24]. Study conducted on QOL among garment workers found that environmental domain was affected followed by social, physical and psychological domain [25]. Study among packing workers found that QOL was affected mainly on physical domain followed by all the other domains [26]. 

The study has certain limitations, including its focus on single district and relatively small sample size, which was influenced by limited manpower and financial constraints.

## Conclusion

The study specifically identified health issues pertaining to the nature of work. Cashew workers are predominantly at high risk for ergonomic, physical, psychological and chemical health hazards. Musculoskeletal pain is a commonly reported health concern. Furthermore, an unsatisfactory working environment and insufficient and improper use of personnel protective equipment exacerbate health problems. However, quality of life is affected by the above health problems, in addition to financial burdens due to low socioeconomic status. To improve the health of workers, implementing safety measures such as installing exhaust fans, providing adequate rest time, ensuring proper sitting postures, and offering medical facilities within the units would be helpful. Frequent health education sessions and frequent health checks once every 3 months would be beneficial. Ergonomic training should be given to the workers before being employed. Personal protective equipment must be provided to all workers, who are encouraged to use it despite their ease of doing work without using it.
